# Spatial and Temporal Evolution of Bluetongue Virus in Wild Ruminants, Spain

**DOI:** 10.3201/eid1406.071586

**Published:** 2008-06

**Authors:** Francisco Ruiz-Fons, Álvaro R. Reyes-García, Vicente Alcaide, Christian Gortázar

**Affiliations:** * Instituto de Investigación en Recursos Cinegéticos, Ciudad Real, Spain; † Macaulay Land Use Research Institute, Aberdeen, UK; ‡ Centro de Investigación en Sanidad Animal El Chaparrillo, Ciudad Real

**Keywords:** Epidemiology, Spain, Europe, Orbivirus, bluetongue virus, vector-borne diseases, wildlife, ruminants, dispatch

## Abstract

We confirmed the emergence of bluetongue virus (BTV) in 5 wild ruminant species in Spain. BTV seroprevalence was high and dispersed with time, with a south-to-north gradient. Our results suggest a complex epidemiology of BTV and underline the need for additional research on wildlife in Europe.

In October 2004, an outbreak of bluetongue caused by bluetongue virus serotype 4 (BTV-4) occurred in southern Spain (www.oie.int/hs2/zi_pays_mald.asp?c_pays=58&c_mald=10&annee=2004). Since then, several BTV-4 outbreaks have occurred in livestock in Spain. Recently, BTV-1 emerged in Spain and caused several outbreaks in livestock (www.oie.int/wahid-rod/public.php?page=single_report&pop=1&reportid=5799). In addition, BTV-8 emerged in central Europe in 2006 (*1*) (www.oie.int/esp/press/es_061023.htm). New outbreaks of BTV-8 occurred in 2007, and this virus serotype was recently detected in the United Kingdom (www.oie.int/wahid-prod/public.php?page=disease_immediate_summary).

BTV is a vector-borne pathogen; *Culicoides* species biting midges (Diptera: Ceratopogonidae) are its biologic vectors (*2*). The main BTV vector in Europe is *C*. *imicola* (*2*), a seasonal species that appears from late May to November (*3*). Other *Culicoides* species may also be vectors of BTV in Europe (*4**,**5*; www.oie.int/esp/press/es_061023.htm). BTV is distributed worldwide between the latitudes 42°30′N and 35°S (*6*), but it has recently spread northward (*1*,*7*). The potential for economic losses make BT a disease reportable to the World Organisation for Animal Health.

Several wild ruminant species are susceptible to BTV infection (*8*–*10*). Red deer (*Cervus elaphus*) and roe deer (*Capreolus capreolus*) are the most common wild ruminants in Spain; mouflon (*Ovis aries*), fallow deer (*Dama dama*), and aoudad (*Ammotragus lervia*) are less common (*11*). The distribution and density of wild ungulates have increased in recent decades in Spain (*12*), but the role of European wild ruminants in the epidemiology of BTV is unknown.

When diseases are transmitted between wildlife and livestock, wildlife disease research is an important tool in establishing effective disease control programs. The risk of wildlife acting as BTV reservoirs and relevant epidemiologic factors need to be explored (*13*). The situation in Spain, with well-distributed wild ruminant species and the presence of BTV vectors and BTV-4, led us to study the status of these ruminants in the epidemiology of BTV.

## The Study

The study area included the southern half of peninsular Spain. This area was selected on the basis of the distribution of *C*. *imicola* and the geographic distribution of BTV-4 outbreaks in livestock and the associated animal movement restriction area (www.mapa.es).

**Figure 1:**
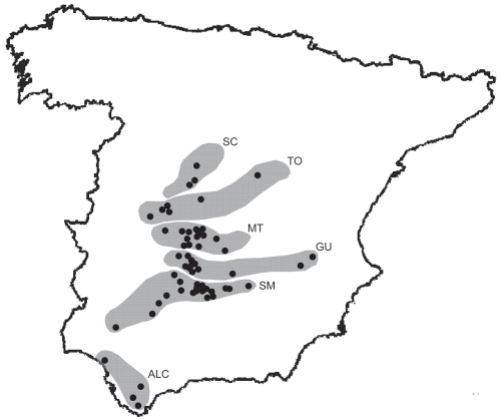
Map of Spain showing wild ruminant sampling sites (black dots) and areas (gray) sampled along a south-to-north gradient during 2003–2007. SC, Sistema Central; TO, Toledo Province; MT, Montes de Toledo; GU, Guadiana Valley; SM, Sierra Morena; ALC, Alcornocales.

Blood samples were obtained from 2,233 red deer, 106 fallow deer, 44 roe deer, 72 mouflon, and 10 aoudad during 2003–2007 in 62 locations (Figure 1). Most (n = 1,575) of the red deer samples and all samples from other ruminant species were collected from hunted animals; some samples (n = 658) from red deer were collected on 5 farms. These farms were located in the Alcornocales (ALC), Sierra Morena (SM), Guadiana Valley (GU), Montes de Toledo (MT), and Sistema Central (SC) areas. Most (69%) samples were collected during the hunting season (October–February). Samples were not obtained during certain periods because of logistic surveillance constraints (online Appendix Table, available from www.cdc.gov/EID/content/14/6/951-appT.htm). Sex and age of red deer were determined, the latter according to tooth eruption patterns (*14*). Animals <1 year of age were classified as juveniles, those 1–2 years of age as subadults, and those >2 years of age as adults.

Blood was collected into sterile tubes from the heart or thoracic cavity from hunted animals and by cervical puncture from farm animals. Samples were centrifuged, and serum was stored at –20°C. Antibodies to BTV in sera were detected by using a competitive ELISA (Pourquier ELISA Bluetongue Serum; Institut Pourquier, Montpellier, France) according to manufacturer’s instructions. The inhibition value of the ELISA for domestic ruminants has been found to be applicable for testing serum from wild ruminants (*15*).

We expected a south-to-north gradient in the expansion of BTV across Spain as observed in livestock. Thus, sampling sites were grouped into 6 areas according to a south-to-north gradient (Figure 1). These areas included southern (ALC), south-central (SM, GU, and MT), and central (Toledo Province and SC) Spain. On the basis of annual distribution of BT outbreaks in Mediterranean countries (*2*), a year was defined as the period July–June.

None of the samples collected in 2003–2004 and 2004–2005 had antibodies to BTV; antibody-positive samples were detected in 2005–2006 and 2006–2007. Mean seroprevalence values for ruminant species analyzed are shown in the Table. A total of 17% ±2% of red deer sampled in 2005–2006 and 29% ±4% of those sampled in 2006–2007 had antibodies to BTV. Northernmost red deer (SC area) were antibody positive only in 2006–2007. However, the number of samples (n = 5) tested in this area the previous year was low.

**Table:** Seroprevalence of bluetongue virus in wild ruminant species, Spain, 2005–2007*

Area	Red deer			Fallow deer			Roe deer			Mouflon			Aoudad	
	n/N	% (SE)		n/N	% (SE)		n/N	% (SE)		n/N	% (SE)		n/N	% (SE)
ALC	116/187	62 (0.04)		25/64	39.1 (0.06)		1/35	2.9 (0.03)		ND	ND		ND	ND
SM	111/580	19.1 (0.02)		9/17	52 (0.12)		ND	ND		0/1	0		ND	ND
GU	36/186	19.4 (0.03)		0/1	0		1/4	25 (0.22)		1/22	4.5 (0.04)		1/4	25 (0.22)
MT	34/435	7.8 (0.01)		0/14	0		ND	ND		8/42	19.1 (0.06)		ND	ND
TO	ND	ND		ND	ND		ND	ND		ND	ND		ND	ND
SC	12/21	57.1 (0.11)		ND	ND		ND	ND		0/3	0		ND	ND
Total	309/1,409	21.9 (0.01)		34/96	35.4 (0.05)		2/39	5.1 (0.04)		9/68	13.2 (0.04)		1/4	25 (0.22)

*n/N, no. positive/no. tested; SE, standard error; ALC, Alcornocales; ND, not determined; SM, Sierra Morena; GU, Guadiana Valley; MT, Montes de Toledo; TO, Toledo Province; SC, Sistema Central.

**Figure 2:**
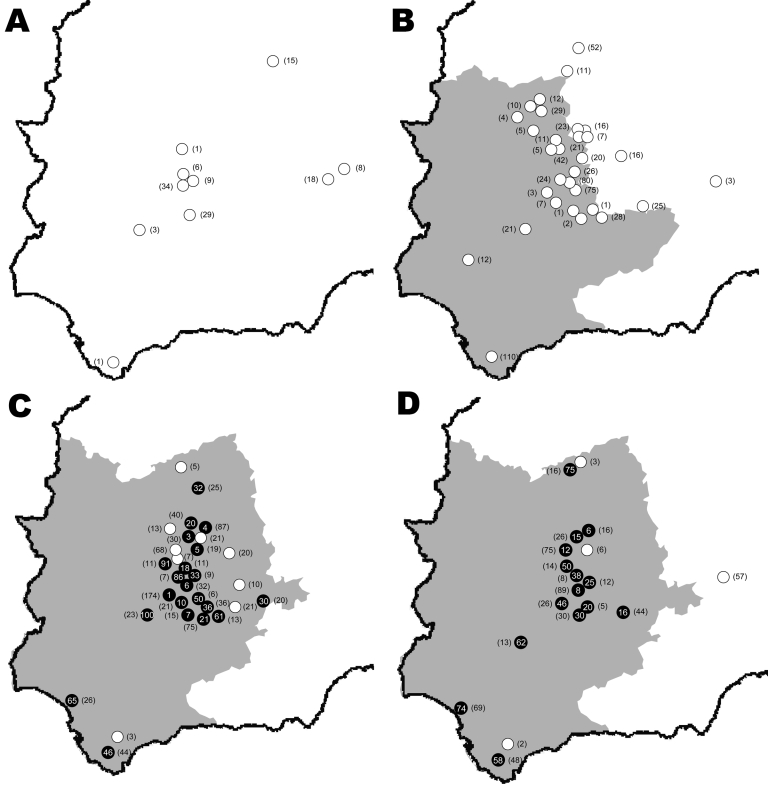
Study areas in Spain showing wild ruminant sampling sites by study year. A) 2003–2004; B) 2004–2005; C) 2005–2006; D) 2006–2007. A year is defined as the period July–June. Animal movement restriction areas for each year (www.mapa.es) are shown (gray areas). White dots show wild ruminant seronegative sampling sites, and black dots show level of bluetongue virus seroprevalence in wild ruminants. Numbers of sampled wild animals per sampling site are shown in parentheses.

BTV seroprevalence in red deer decreased along a south-to-north gradient and increased throughout the years sampled (online Appendix Table, available from www.cdc.gov/EID/content/14/6/951-appT.htm and Figure 2). Mean seroprevalence was slightly higher in males and adults but dependent on the sampling area (online Appendix Table, available from www.cdc.gov/EID/content/14/6/951-appT.htm). Because the first evidence of contact with BTV occurred in 2005, observed seroprevalences can be considered as incidence rates. Thus, in 2005–2006, the incidence rate was higher in subadults than in adults and juveniles.

## Conclusions

The presence of antibodies to BTV in wild ruminants has recently been studied in Belgium (A. Linden, pers. comm.) and Germany (www.efsa.europa.eu/EFSA/DocumentSet/appx6_bluetongue_S8_en.pdf). In 2006, BTV seroprevalence was 0.58% in Belgian cervids. The survey in Germany showed BTV seroprevalence rates of 0.09% in red deer, 5.7% in roe deer, and 4.9% in mouflon. Our study provides evidence that BTV is present in wild ruminants in Europe over a large area.

All ruminant species studied were positive for BTV. This result is not surprising because other wild ruminant species have been reported to be susceptible to BTV infection (*8*–*10*). We observed some differences in BTV seroprevalence between ruminant species (Table). However, if one considers the epidemic nature of BTV, differences in the number of seropositive ruminant species, and the unequal distribution of *C*. *imicola* in the study area (*2*; www.mapa.es), the observed results may be caused by differences in susceptibility to the vector/pathogen or differences in vector/pathogen distribution across the study area.

We observed similar spatial and temporal BTV patterns in red deer (Figure 2) and livestock. Nevertheless, we found the first evidence of contact with BTV 1 year later in red deer than in livestock. This delay may have been caused by larger numbers of samples from livestock than from wild ruminants. However, our findings suggest that wild ruminants, particularly cervids because of their wider distribution in Europe, could be used as sentinels for surveillance of BTV. Moreover, the high BTV seroprevalence in cervids from the southernmost sampling area suggests that cervids may not interfere with vaccinations given in this region.

This study shows an increased distribution of BTV across Spain and that wild ruminants in Europe can be infected with BTV. Our findings, combined with those of earlier studies, suggest a complex epidemiologic scenario of BTV in Europe with many susceptible hosts, an increase in its main vector because of climate changes, and the appearance of new competent vectors. Nevertheless, more information on the role of susceptible wild ruminant species is needed to clarify the complexity of BTV epidemiology in Europe.

## References

[1] Bluetongue in northern Europe: serotype identified. Vet Rec. 2006;159:294.

[2] Mellor PS, Wittmann EJ. Bluetongue virus in the Mediterranean Basin 1998–2001. Vet J. 2002;164:20–37. 10.1053/tvjl.2002.0713 12359482

[3] Ortega MD, Mellor PS, Rawlings P, Pro MJ. The seasonal and geographical distribution of *Culicoides imicola, C. pulicaris* group and *C. obsoletus* group biting midges in central and southern Spain. Arch Virol Suppl. 1998;14:85–91. 9785498 10.1007/978-3-7091-6823-3_9

[4] Caracappa S, Torina A, Guercio A, Vitale F, Calabrò A, Purpari G, Identification of a novel bluetongue virus vector species of *Culicoides* in Sicily. Vet Rec. 2003;153:71–4. 12892265 10.1136/vr.153.3.71

[5] De Liberato C, Scavia G, Lorenzetti R, Scaramozzino P, Amaddeo D, Cardeti G, Identification of *Culicoides obsoletus* (Diptera: Ceratopogonidae) as a vector of bluetongue virus in central Italy. Vet Rec. 2005;156:301–4. 15786918 10.1136/vr.156.10.301

[6] Mellor PS, Boorman J, Baylis M. *Culicoides* biting midges: their role as arbovirus vectors. Annu Rev Entomol. 2000;45:307–40. 10.1146/annurev.ento.45.1.307 10761580

[7] Toussaint JF, Sailleau C, Mast J, Houdart P, Czaplicki G, Demeestere L, Bluetongue in Belgium, 2006. Emerg Infect Dis. 2007;13:614–6. 17553280 10.3201/eid1304.061136 PMC2725968

[8] Trainer DO, Jochim MM. Serologic evidence of bluetongue in wild ruminants of North America. Am J Vet Res. 1969;30:2007–11. 4310434

[9] Tessaro SV, Clavijo A. Duration of bluetongue viremia in experimentally infected American bison. J Wildl Dis. 2001;37:722–9. 11763735 10.7589/0090-3558-37.4.722

[10] Fischer-Tenhagen C, Hamblin C, Quandt S, Frölich K. Serosurvey for selected infectious disease agents in free-ranging black and white rhinoceros in Africa. J Wildl Dis. 2000;36:316–23. 10813614 10.7589/0090-3558-36.2.316

[11] Palomo LJ, Gisbert J, eds. Atlas de los mamíferos terrestres de España. Madrid (Spain): Dirección General de Conservación de la Naturaleza-SECEM-SECEMU; 2002.

[12] Acevedo P, Delibes-Mateos M, Escudero MA, Vicente J, Marco J, Gortazar C. Environmental constraints in the colonization sequence of roe deer (*Capreolus capreolus* Linnaeus, 1758) across the Iberian Mountains, Spain. J Biogeogr. 2005;32:1671–80. 10.1111/j.1365-2699.2005.01310.x

[13] Ruiz-Fons F, Segalés J, Gortázar C. A review of viral diseases of the European wild boar: effects of population dynamics and reservoir role. [Epub ahead of print]. Vet J. 2007; (Apr):7. 17420149 10.1016/j.tvjl.2007.02.017 PMC7110567

[14] Saenz de Buruaga M, Lucio AJ, Purroy J. Reconocimiento de sexo y edad en especies cinegéticas. Vitoria-Gasteiz (Spain): Diputación Foral de Álava; 1991.

[15] Afshar A, Heckert RA, Dulac GC, Trotter HC, Myers DJ. Application of a competitive ELISA for the detection of bluetongue virus antibodies in llamas and wild ruminants. J Wildl Dis. 1995;31:327–30. 8592352 10.7589/0090-3558-31.3.327

